# Supplementary material for: APOBEC3A is an oral cancer prognostic biomarker in Taiwanese carriers of an APOBEC deletion polymorphism
Source: Nat Commun. 2017 Sep 6;8:465. doi: 10.1038/s41467-017-00493-9 (PMC5587710; doi:10.1038/s41467-017-00493-9)
Supplement: Supplementary file 1 — Supplementary Information [file 41467_2017_493_MOESM1_ESM.pdf]

Description of Supplementary Files

File Name: Supplementary Information
Description: Supplementary Figures, Supplementary Tables and Supplementary References

File Name: Supplementary Data 1
Description: Statistics of WES for 50 T/N paired samples.

File Name: Supplementary Data 2
Description: Statistics of RNA-Seq for 39 T/N paired samples.

File Name: Supplementary Data 3
Description: Mutations identified from WES with Mutect.

File Name: Supplementary Data 4
Description: Mutations identified from CCP with Mutect.

File Name: Supplementary Data 5
Description: Significantly amplified regions from 50 WXS (OSCC-Taiwan).

File Name: Supplementary Data 6
Description: Significantly deleted regions from 50 WXS (OSCC-Taiwan).

File Name: Supplementary Data 7
Description: Enrichment analysis result for the 3,548 DEGs.

File Name: Supplementary Data 8
Description: Number of reads that support A3B deletion from WES or RNA-Seq.

File Name: Peer Review File

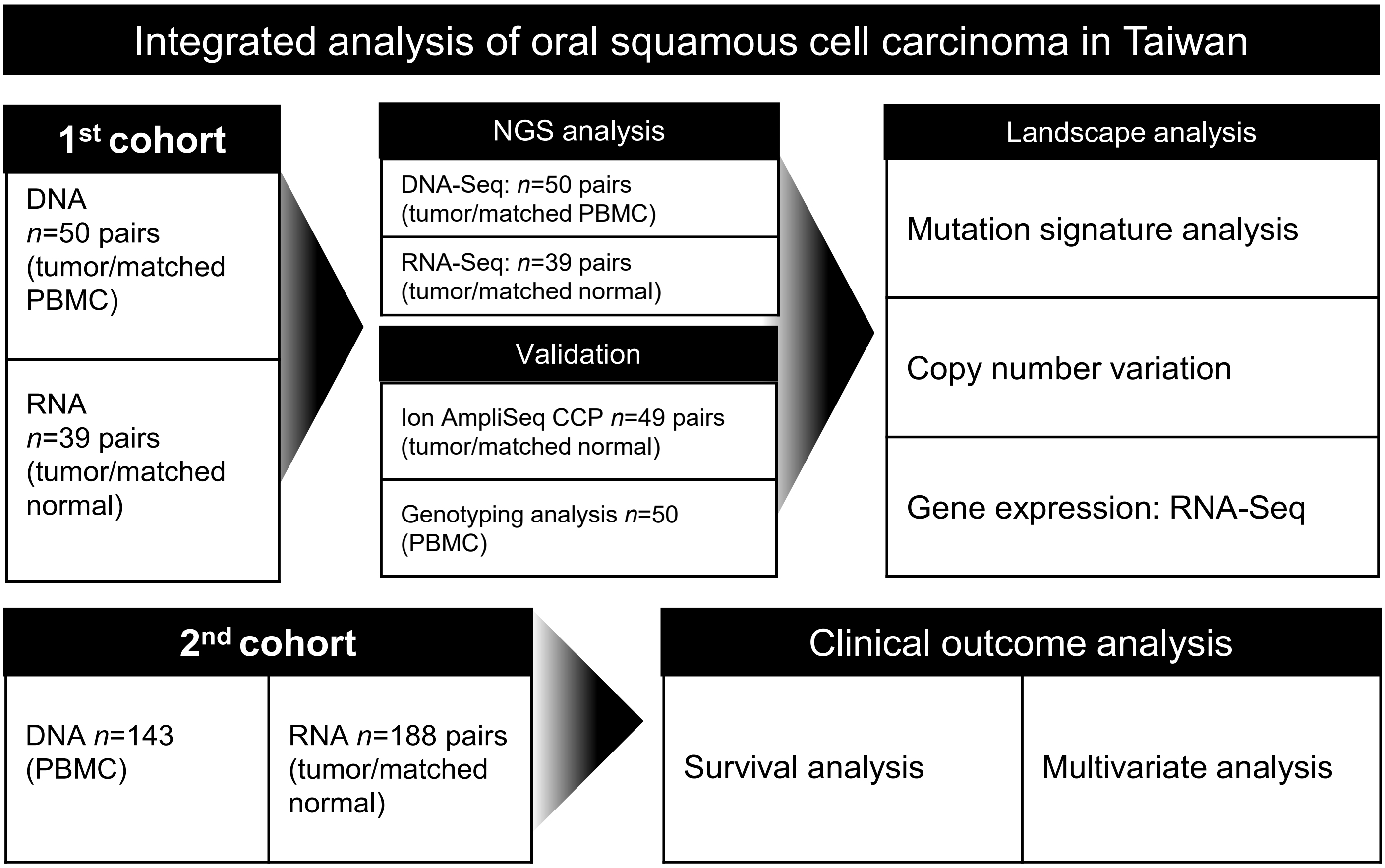

Supplementary Figure 1. Flowchart summary of the experimental design and step-wise analyses employed in this study. In this study, two independent cohorts were used for discovery (upper) and validation (bottom). There were a total of 50 OSCC patients in the 1st cohort (Supplementary Table S1). WES were carried out for all 50 pairs of tumor tissues and matched PBMC cells and RNA-Seq were carried out for 39 paired tumor/matched normal tissues. We validated a subset of SNVs found in WES with Ion AmpliSeq comprehensive cancer panel (CCP) for 49 paired tumor/matched PBMC samples. *A3B* deletion polymorphism in the first cohort was verified by PCR-based genotyping analysis. Mutation signatures, copy number variations and expression profiles were analyzed on the basis of WES and RNA-Seq data. To further confirm the upregulation of *A3A* and *A3B* expression in OSCC samples and inspect the consequence of this expression alteration, we further included another 188 OSCC patients, from which we obtained RNA from 188 paired tumor/normal matched tissues and 143 PBMC DNA samples. We then examined the relationship between the clinical outcome and expression levels of *A3A* and *A3B*.

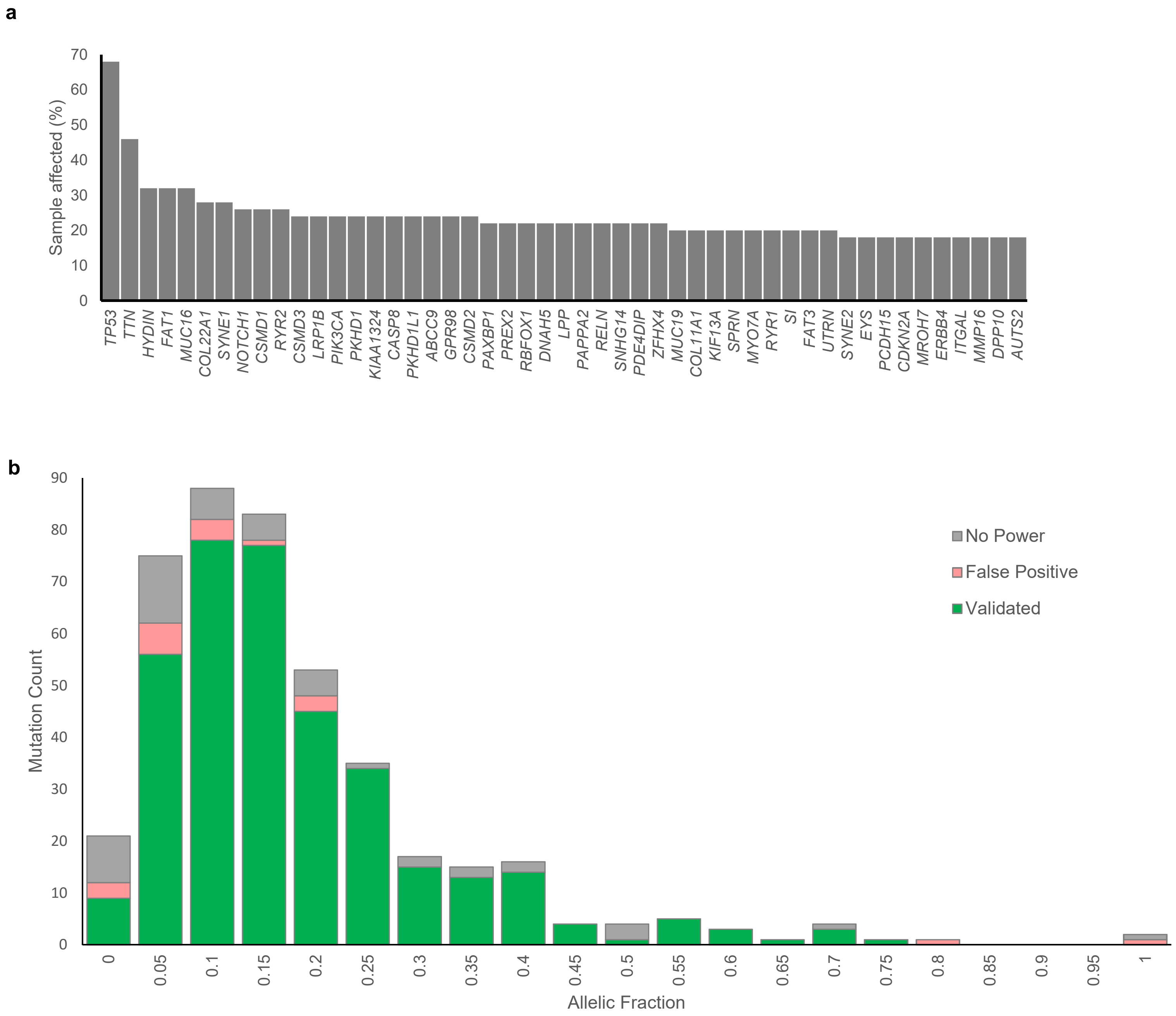

Supplementary Figure 2. Top 50 frequently mutated genes in OSCC-Taiwan and independent validation of identified mutations by CCP. (a) Genes most likely to be altered by the mutations in tumor samples are ranked by mutation incidence. Y-axis denotes the percentages (%) of patients with the indicated mutated genes found in their tumor samples. This figure shows effected genes without applying variant allelic fraction filters. (b) For SNVs identified by the WES approach, we used CCP to validate mutations that are covered by the panel. The bar graph illustrates the extent of validation as well as the distribution of validated mutations according to allelic frequency. Confirmed (Validated) and non-confirmed (False Positive) sites are respectively represented by green and pink bars. Site without adequate coverage for validation with 95% power are denoted in gray (No Power). The X- axis denotes the allelic fraction as determined by WES, while the Y-axis indicates the counts of CCP- covered mutations.

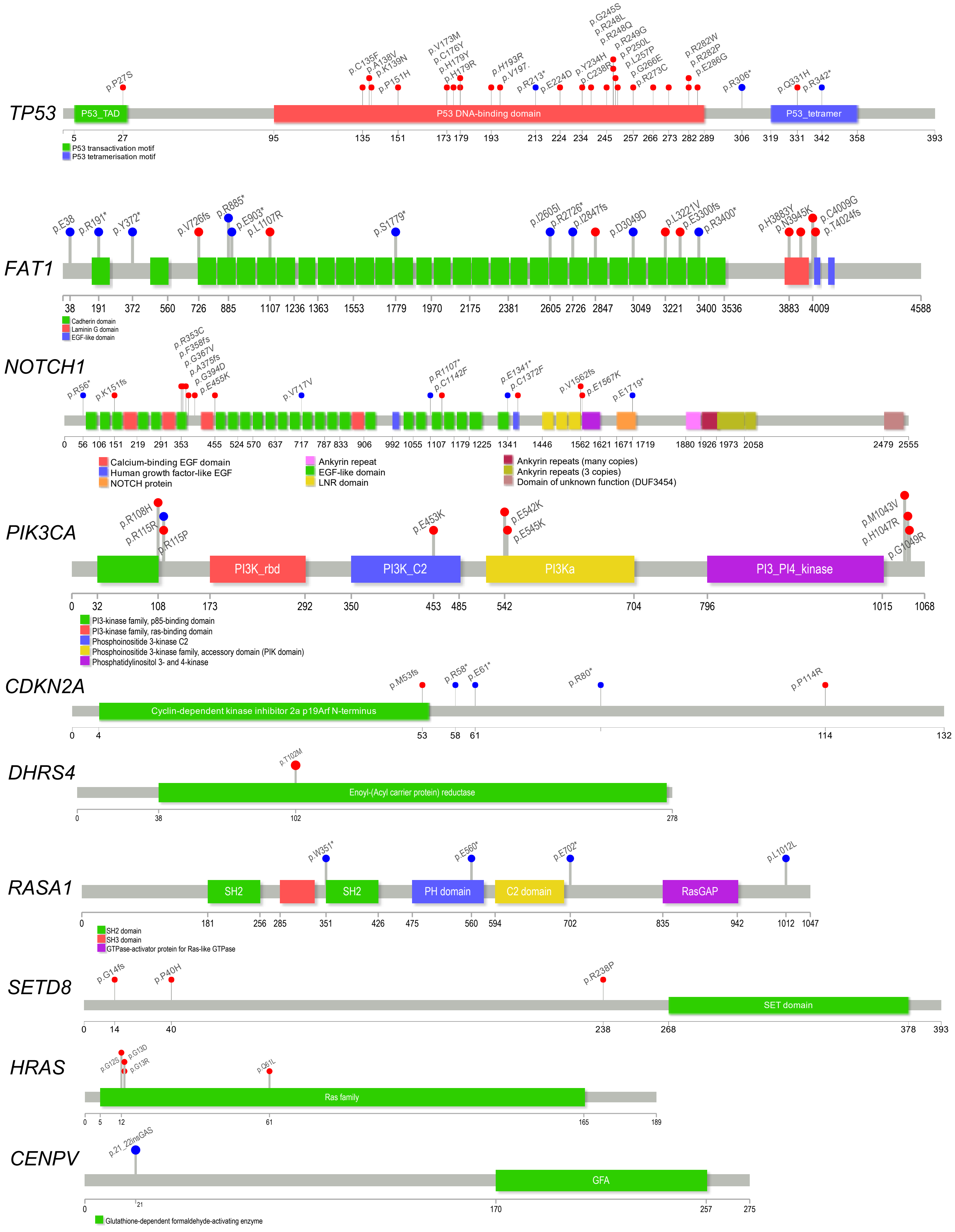

Supplementary Figure 3. Predicted coding impact by transcript base position and functional domain for mutated genes. The lollipop diagrams represent the transcript base change, and the rectangle boxes with different color represent the functional domains. Synonymous mutations are shown by blue circle; nonsynonymous mutations are shown by red circle.

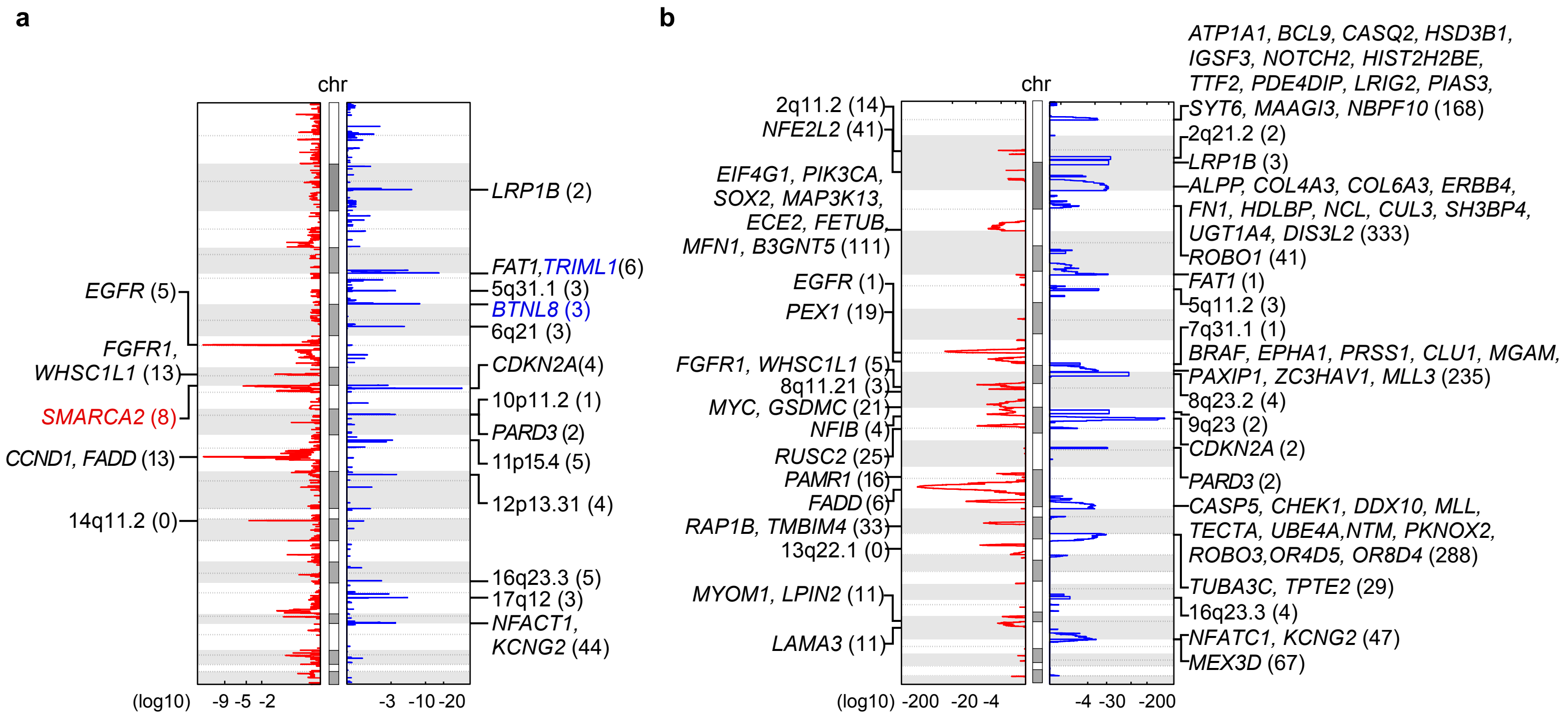

Supplementary Figure 4. Copy number variations identified in (a) OSCC-Taiwan and (b) OSCC-TCGA. Regions with significantly overrepresented amplification (left) or deletion (right) were listed together with their residual q values; only regions with q values of less than 0.5 are listed. Potential driver genes within these focal alterations are listed, together with the numbers of resident genes in the corresponding peaks (in parentheses). The novel copy number amplified or deleted driver genes (*SMARCA2, TRIML1* and *BTNL8*) are marked in red and blue, respectively.

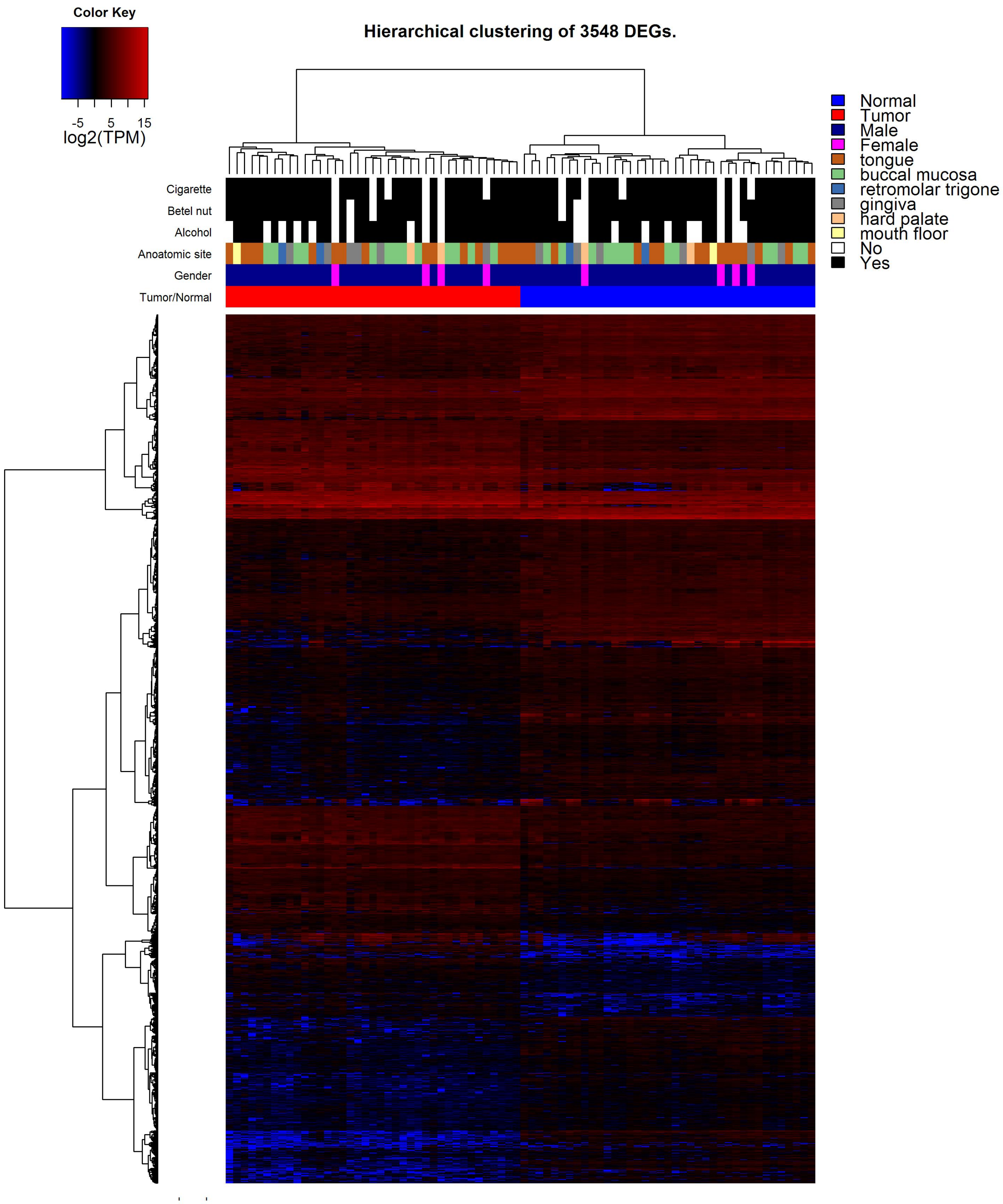

Supplementary Figure 5. Hieratical clustering of normalized read counts for the 3,548 DEGs identified in RNA-Seq. Demographic information is shown in rows below the top dendrogram. Unsupervised clustering of these DEGs led to separation of the 39 normal and 39 tumor samples into two main branches.

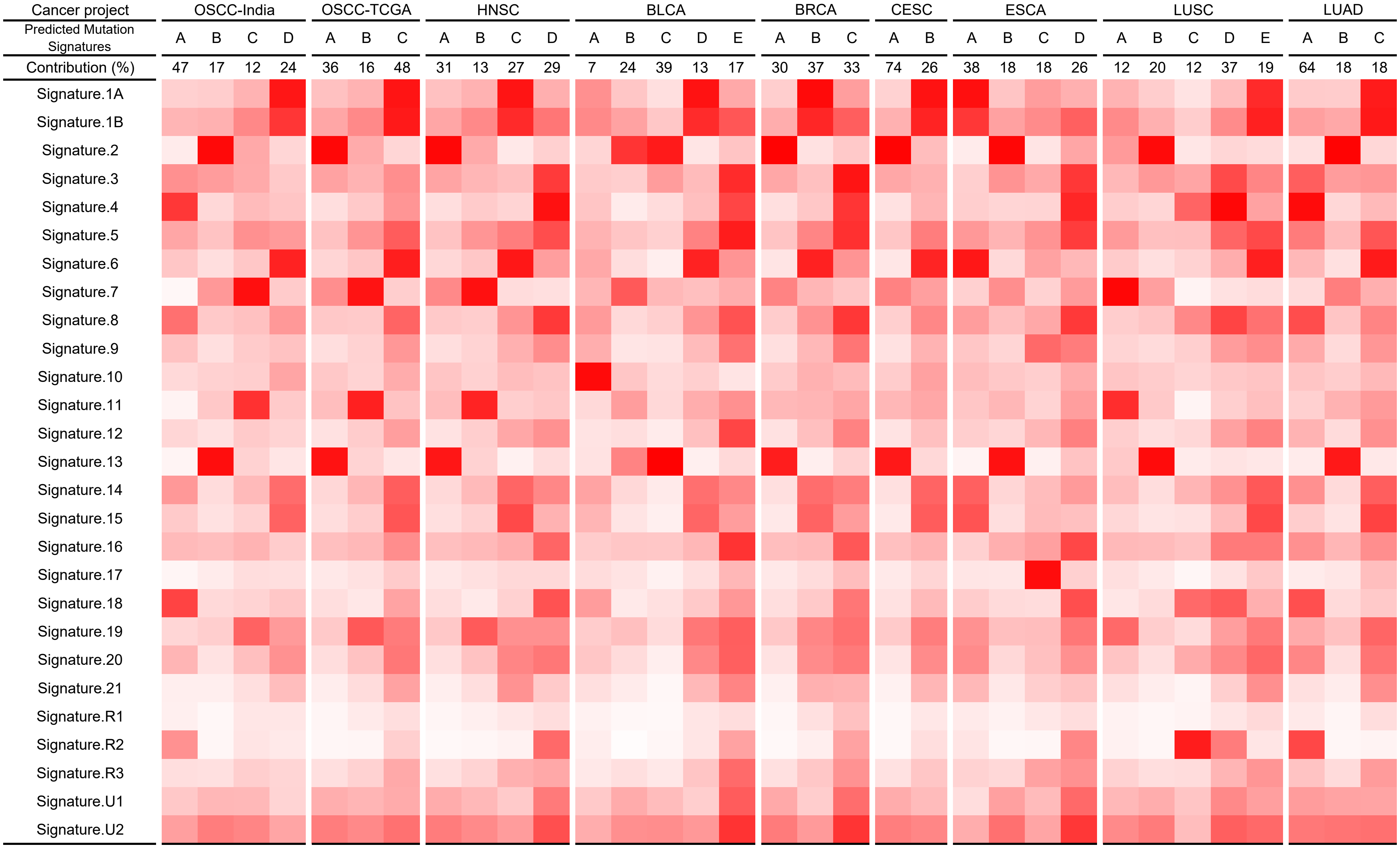

Supplementary Figure 6. Cosine similarity for mutational signatures found in cancers reported to have APOBEC-associated mutation signature. After mutation signatures were identified, cosine similarity was used to represents the extent of similarity to particular signatures as defined previously (see on-line Methods). The heatmap depicts cosine similarity result (score ranging from 0 to 1) on the mutation spectrums of OSCC-India, OSCC-TCGA, HNSC, BLCA, BRCA, CESC, ESCA, LUSC, and LUAD. OSCC-TCGA, a subset of OSCC from HNSC in TCGA. HNSC, head and neck squamous cell carcinoma. BLCA, bladder urothelial carcinoma. BRCA, breast invasive carcinoma. CESC, cervical squamous cell carcinoma. ESCA, esophageal carcinoma. LUSC, lung squamous cell carcinoma. LUAD, lung adenocarcinoma.

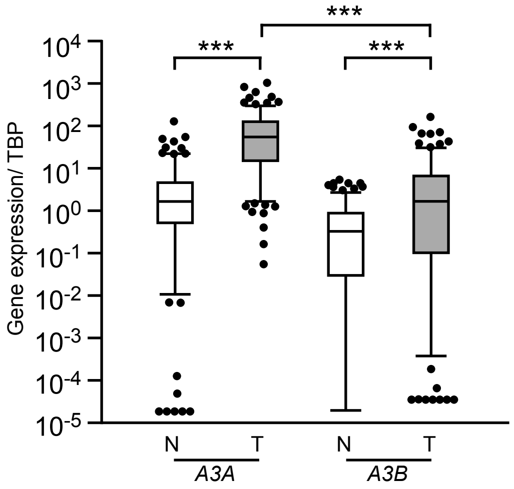

Supplementary Figure 7. Expression level of *A3A* and *A3B* in the 2nd cohort. A total of 188 paired OSCC clinical samples were subjected to qRT-PCR analysis. Relative gene expression levels of *A3A* and *A3B* were both normalized to that of the housekeeping gene, *TBP* (TATA box binding protein). Expression levels of *A3A* and *A3B* were both significantly upregulated in OSCC ($p < 0.001$). Expression level of *A3A* was further higher than that of *A3B* in tumors ($p < 0.001$). Box plots show the distribution of expression of indicated APOBEC genes. Boxes extend from the third (Q3) to the first (Q1) quartile, with the line at the median; whiskers extend to 5 and 95 percentiles.

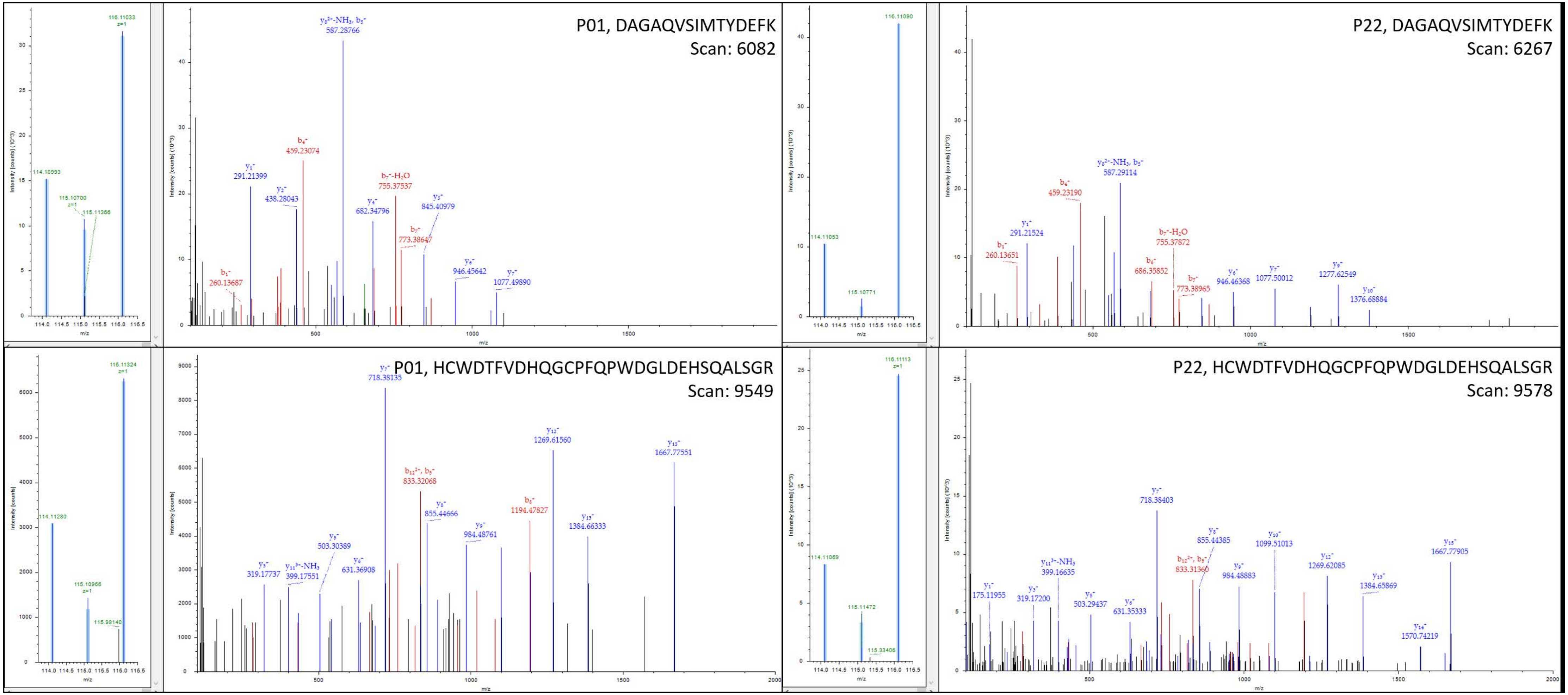

Supplementary Figure 8. A3A protein identification and quantification by LC-MS/MS analysis of OSCC tumors. Each panel shows both low mass reporter ion of iTRAQ reagent (114, 115 and 116) and MS/MS spectrum assigned to unique peptides of A3A in patient #1 (left 2 panels) and patient #22 (right 2 panels).

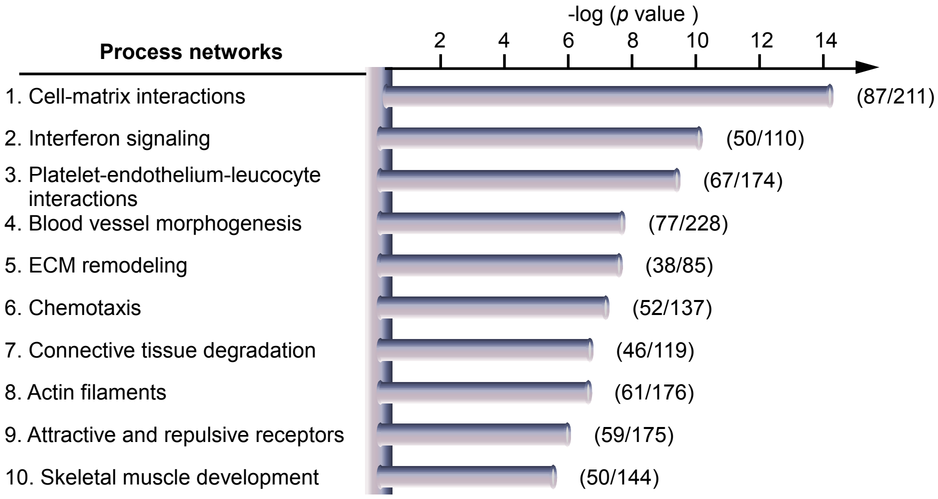

Supplementary Figure 9. Pathways that are enriched with differentially expressed genes found in the tumor samples of OSCC-Taiwan. Notably, inflammatory interferon signaling network ranked second among the significantly enriched processes.

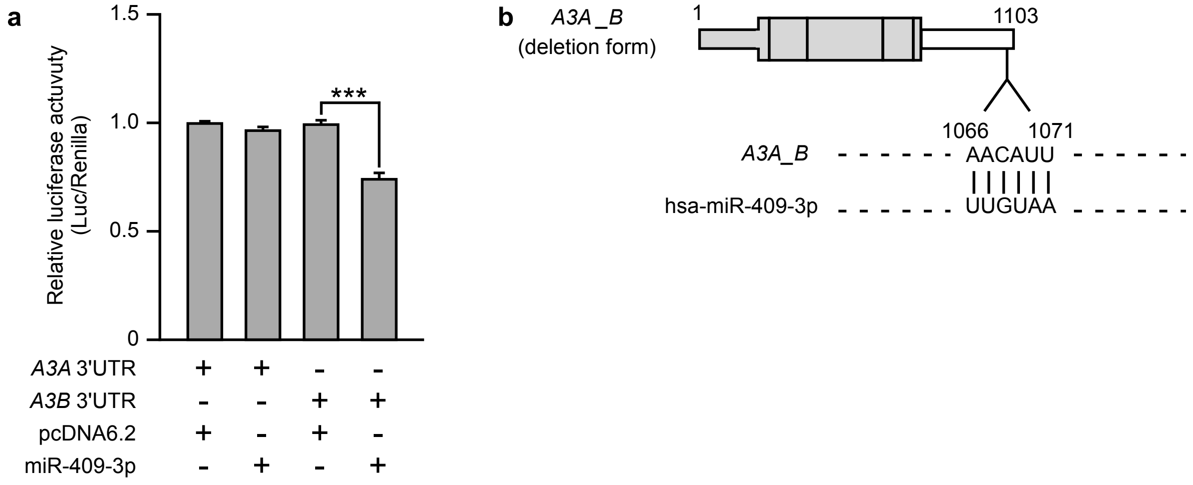

Supplementary Figure 10. Potential miRNA regulation of *A3B* 3'UTR. (a) Luciferase reporter assay demonstrated that miR-409 may target the *A3B* 3'UTR but not *A3A* 3'UTR. Comparing to vector control (pcDNA6.2), ectopic miR-409 led to 20% reduction of luciferase activity (error bars: s.d.; $p < 0.001$). (b) Schematic depiction of the predicted target site of miR-409 in the 3'UTR of *A3A_B*.

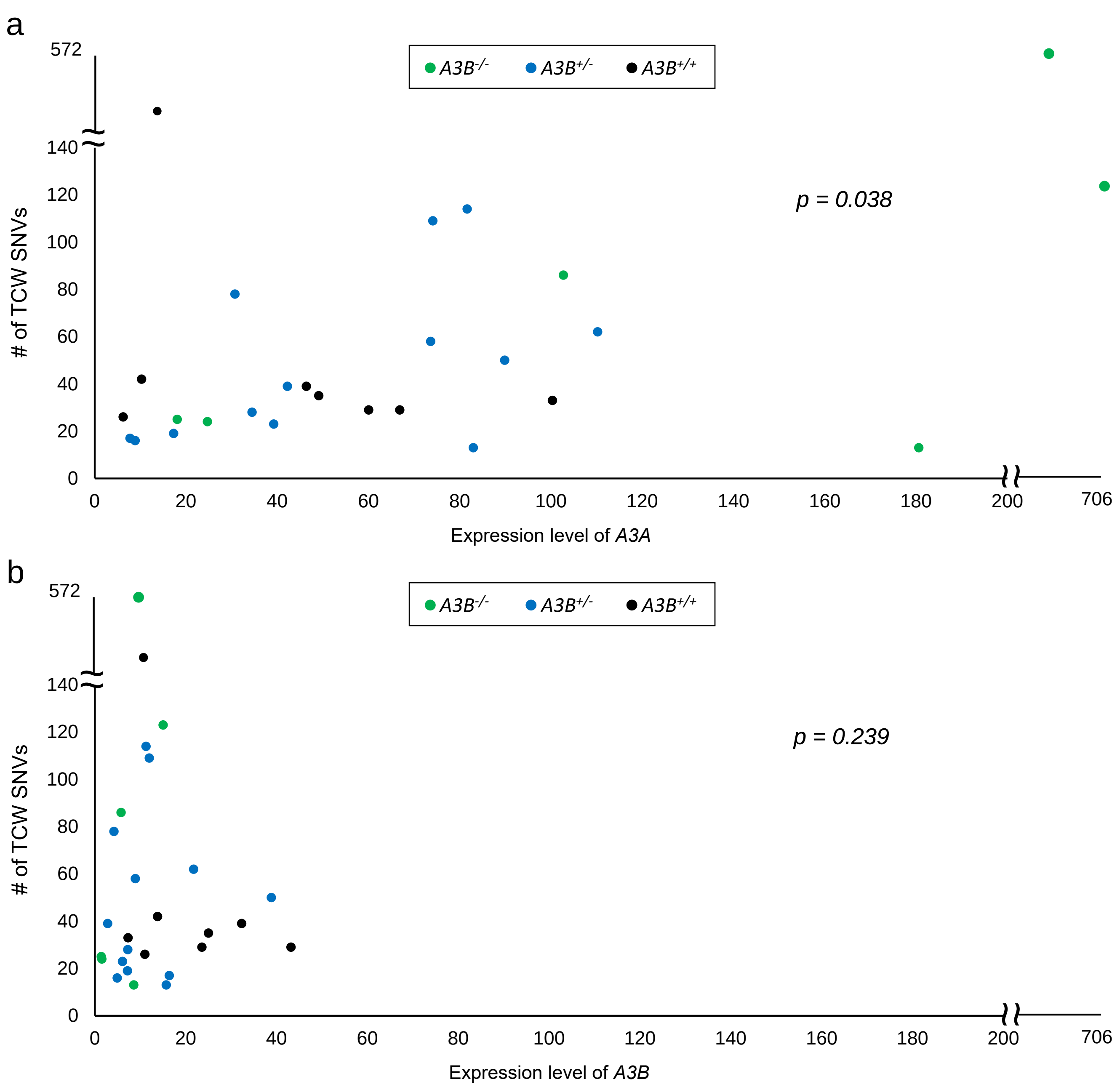

Supplementary Figure 11. Correlation between APOBEC-associated single nucleotide variations (SNVs) and expression level of *A3A* and *A3B*. The Y-axis represents the number of somatic TCW (C>T, C>G) mutations, while the X-axis shows the expression level of (a) *A3A* and (b) *A3B* on the basis of TPM (transcripts per million). The p values were calculated with Spearman's rank correlation. The dots colored by green represent $A3B^{-/-}$, the dots colored by blue represent $A3B^{+/-}$, and the dots colored by black represent $A3B^{+/+}$.

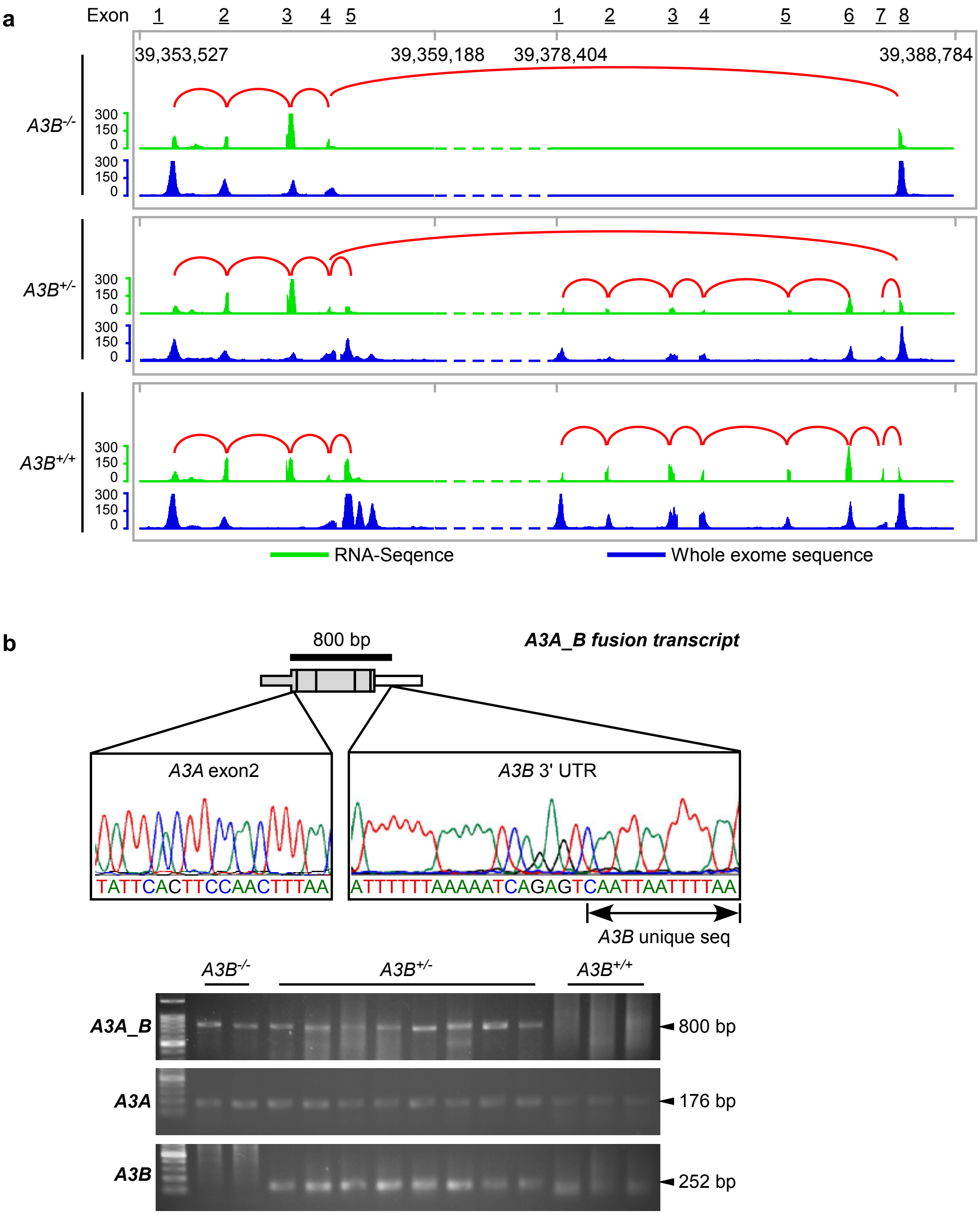

Supplementary Figure 12. Detection of fused gene *A3A_B* in WES, RNASeq and RT-PCR. (a) Representative distribution of RNA-Seq and WES reads in the APOBEC3 locus from $A3B^{-/-}$ (top), $A3B^{+/-}$ (middle), and $A3B^{+/+}$ (bottom) samples. Reads for RNA-Seq and WES are shown in green and blue, respectively. Red lines represent reads corresponding to junctional sequences. The height of a peak is proportional to the read number. (b) RT-PCR of the *A3A_B* fusion transcript. The 800 bp RT-PCR product was derived from the *A3A_B* fusion transcript. PCR products were resolved by Sanger sequencing (top) or size (gel electrophoresis, bottom) to distinguish genotype-specific transcript expression.

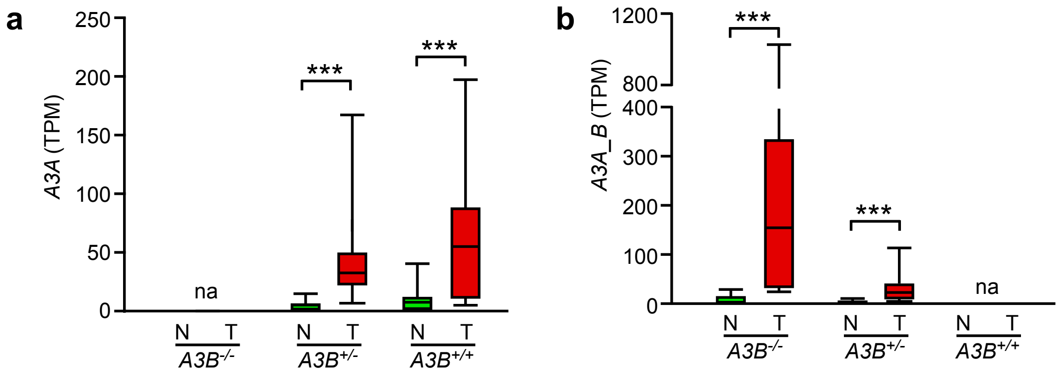

Supplementary Figure 13. Expression levels of *A3A* and *A3A_B* among three genotypes in OSCC tissue samples. Based on RNA-Seq-determined TPM values, the mRNA expression levels of (a) *A3A* and (b) *A3A_B* were determined in the initial cohort of 39 paired samples. Patients are grouped according to their APOBEC-deletion genotypes. Box plots show the distribution of expression of indicated APOBEC genes. Boxes extend from the third (Q3) to the first (Q1) quartile, with the line at the median; whiskers extend to 2.5 and 97.5 percentiles. ($A3B^{-/-}$: n=7; $A3B^{+/-}$: n=20; $A3B^{+/+}$: n=12)

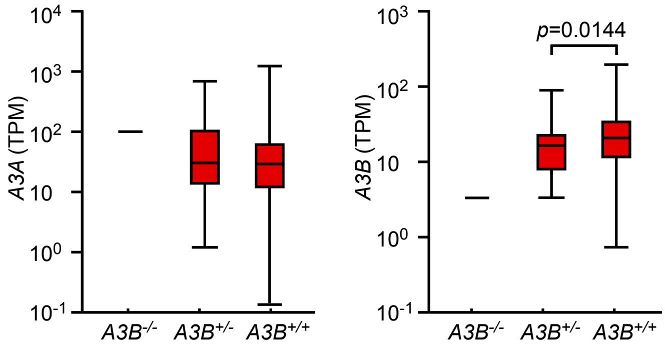

Supplementary Figure 14. Expression levels of *A3A* and *A3B* among three genotypes in OSCC-TCGA samples. No difference and a slight difference were found between the expression levels of *A3A* and *A3B* in *A3B*$^{+/-}$ and *A3B*$^{+/+}$ patients. Box plots show the distribution of expression of indicated APOBEC genes. Boxes extend from the third (Q3) to the first (Q1) quartile, with the line at the median; whiskers extend to 2.5 and 97.5 percentiles. The *p* values were derived from Mann-Whitney test. (*A3B*$^{-/-}$: *n*=1; *A3B*$^{+/-}$: *n*=34; *A3B*$^{+/+}$: *n*=278)

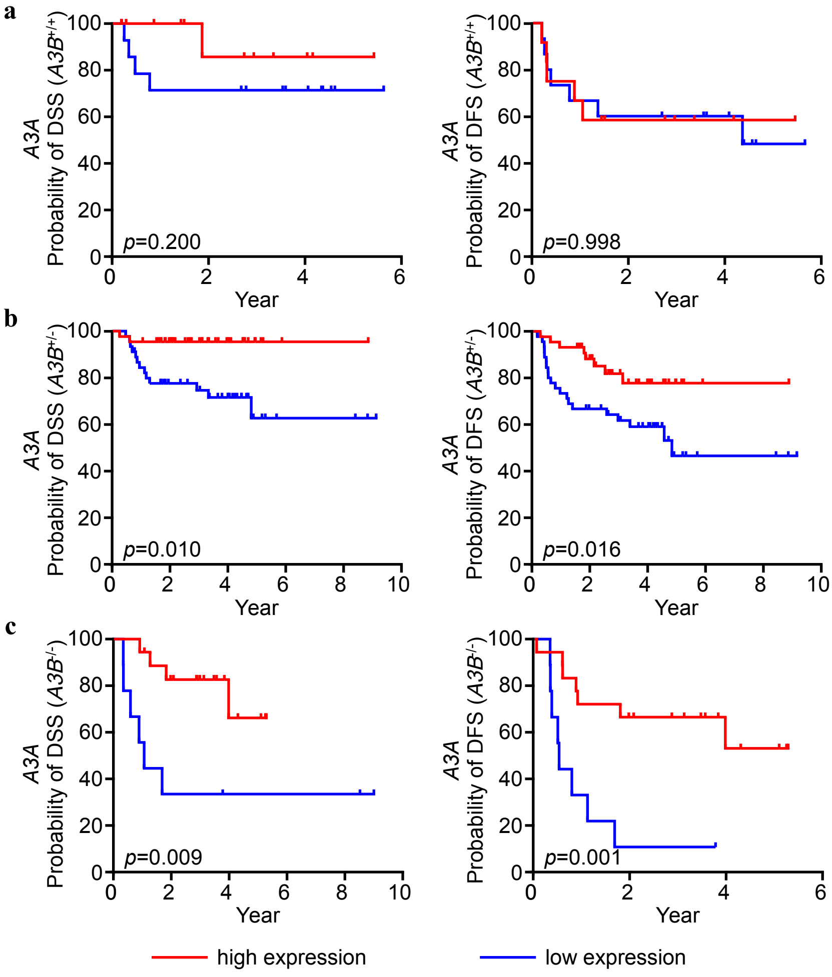

Supplementary Figure 15. Kaplan-Meier plot for disease-specific survival (DSS) and disease-free survival (DFS) by the *A3A* expression level for the 143 patients. (a) No significant difference in DSS and DFS was found for subgroups stratified according to expression level of *A3A* in OSCC patients carrying $A3B^{+/+}$ genotype. On the other hand, high *A3A* in patients with (b) $A3B^{+/-}$ and (c) $A3B^{-/-}$ genotypes, was significantly correlated with better DSS, and DFS. The survival rate was estimated by Kaplan-Meier plotting and compared by log-rank test; all *p* values are two-sided with the significance level set at $p < 0.05$.

Supplementary Tables

Supplementary Table 1. Clinical characteristics for 50 OSCC patients in the 1st cohort

Total patients no.		50
Age (mean ± SD)		52.06 ± 9.87
characteristics		case no. (%)
Gender	Male	44 (88)
	Female	6 (12)
Stage	1	2 (4)
	2	9 (17.64)
	3	4 (8)
	4A	31 (62)
	4B	4 (8)
Differentiation	well	8 (16)
	moderately	34 (68)
	poorly	8 (16)
Site	buccal mucosa	18 (36)
	gingiva	5 (10)
	gum	2 (4)
	hard palate	2 (4)
	mouth floor	1 (2)
	retromolar trigone	3 (6)
	tongue	19 (38)
pN status	0	25 (50)
	1	9 (18)
	2B	14 (28)
	2C	2 (4)
Alcohol drinking	No	17 (34)
	Yes	33 (66)
Betel quid chewing	No	7 (14)
	Yes	43 (86)
Cigarette smoking	No	9 (18)
	Yes	41 (82)
HPV	Positive	4 (8)
	Negative	46(92)

Supplementary Table 2. Significantly altered genes identified with MutSigCV

Gene	q-value	OSCC-Taiwan	OSCC-TCGA	OSCC-India
TP53	<0.0001	68	75	64
FAT1	<0.0001	32	27	31
NOTCH1	<0.0001	26	22	20
CDKN2A	<0.0001	18	25	4
DHRS4	0.065	12	1	0
RASA1	0.113	10	5	3
PIK3CA	0.176	24	17	10
SETD8	0.499	10	1	0
CENPV	0.586	4	0	0
HRAS	0.591	8	5	10

Supplementary Table 3. Clinical characteristics of 188 OSCC patients in the 2nd cohort

**Characteristics**	**OSCC Patient (*n*)**
All cases	188
Gender	
Male	172
Female	16
Age (years)	
Mean ± SD	51.8 ± 10.4
Overall TNM stage	
Stage I	16
Stage II	40
Stage III	20
Stage IV	112
Location	
Buccal	78
Mouth floor	9
Gum	30
Hard palate	6
Lip	7
Tongue	58

Supplementary Table 4. Hypermutation signatures of *A3A* and *A3B* in cancers reported to have APOBEC-associated mutation signature

Project Name	# of Samples	Total Somatic SNP Mutations	TCW	YTCW	RTCW	YTCW(%)	RTCW(%)
OSCC-Taiwan	50	20,963	5,723	3,995	1,728	70%	30%
OSCC-TCGA	172	26,050	6,980	4,658	2,322	67%	33%
OSCC-India	106	13,462	2,319	1,497	822	65%	35%
BLCA-US	130	37,315	17,288	12,252	5,036	71%	29%
BRCA-US	959	78,109	22,164	15,014	7,150	68%	32%
CESC-US	194	44,615	21,855	15,546	6,309	71%	29%
KIRP-US	159	11,786	1,190	684	506	57%	43%
LUSC-US	177	64,339	11,876	7,479	4,397	63%	37%
ORCA-IN	106	13,462	2,319	1,497	822	65%	35%
SKCM-US	335	249,637	45,225	29,654	15,571	66%	34%
STAD-US	288	130,050	6,007	3,316	2,691	55%	45%
UCEC-US	246	181,809	34,977	23,771	11,206	68%	32%

Supplementary Table 5. HPV status checked by PCR and RT-PCR in samples

Sample #	HPV-DNA*	HPV-E6 cDNA
25	type 18	undetectable
49	type 16	undetectable
51	type 16	undetectable
56	type 16	undetectable

*HPV-DNA PCR products were confirmed by Sanger's sequencing.

We tested for HPV DNA in all 50 samples by PCR using genomic DNA, and found 4 cases were positive. However, we did not detect HPV E6 transcript in these 4 cases by RT-PCR using cDNA samples prepared for RNAseq experiments. Three of 4 HPV DNA-positive cases have p53 mutations, typically not related to HPV. This further supported that the mutation signatures in the 50 OSCC cases are not HPV-driven. HPV E6 inactivates p53 through direct protein-protein binding, resulting in deregulation of the cell cycle in HPV-E6 positive cells. Previous reports indicated that the mutation pattern in OSCC tumors without E6 expression is similar to those in HPV-negative tumors[1, 2], while *TP53* gene mutations are inversely correlated with HPV E6 expression[3]. Thus, the mutational signatures observed in the 50 OSCC samples were not driven by HPV.

Supplementary References

1. Wichmann G*, et al.* The role of HPV RNA transcription, immune response-related gene expression and disruptive TP53 mutations in diagnostic and prognostic profiling of head and neck cancer. *Int J Cancer* **137**, 2846-2857 (2015).

2. Braakhuis BJ*, et al.* Genetic patterns in head and neck cancers that contain or lack transcriptionally active human papillomavirus. *J Natl Cancer Inst* **96**, 998-1006 (2004).

3. Westra WH, Taube JM, Poeta ML, Begum S, Sidransky D, Koch WM. Inverse relationship between human papillomavirus-16 infection and disruptive p53 gene mutations in squamous cell carcinoma of the head and neck. *Clin Cancer Res* **14**, 366-369 (2008).
